# Supplementary material for: Effects of Vitamin D Supplementation on COVID-19 Related Outcomes: A Systematic Review and Meta-Analysis
Source: Nutrients. 2022 May 20;14(10):2134. doi: 10.3390/nu14102134 (PMC9147949; doi:10.3390/nu14102134)
Supplement: Supplementary file 1 [file nutrients-14-02134-s001.zip › nutrients-1713929-supplementary.pdf]

Study or Subgroup	Vitamin D supplementation Events	Total	Control Events	Total	Weight	Risk Ratio M-H, Fixed, 95% CI
**Bolus**						
Hosseini et al. 2021	0	18	1	15	0.3%	0.28 [0.01, 6.43]
**Subtotal (95% CI)**		**18**		**15**	**0.3%**	**0.28 [0.01, 6.43]**
Total events	0		1			
Heterogeneity: Not applicable						
Test for overall effect: Z = 0.80 (P = 0.43)						
**No Bolus**						
Meltzer et al, 2021	17	89	14	80	2.3%	1.09 [0.58, 2.07]
Ma et al, 2021	49	363	1329	7938	18.2%	0.81 [0.62, 1.05]
Oristrell 2021	236	8076	183511	5848776	79.2%	0.93 [0.82, 1.06]
**Subtotal (95% CI)**		**8528**		**5856794**	**99.7%**	**0.91 [0.82, 1.02]**
Total events	302		184854			
Heterogeneity: Chi² = 1.24, df = 2 (P = 0.54); I² = 0%						
Test for overall effect: Z = 1.61 (P = 0.11)						
**Total (95% CI)**		**8546**		**5856809**	**100.0%**	**0.91 [0.81, 1.02]**
Total events	302		184855			
Heterogeneity: Chi² = 1.78, df = 3 (P = 0.62); I² = 0%						
Test for overall effect: Z = 1.64 (P = 0.10)						
Test for subgroup differences: Chi² = 0.54, df = 1 (P = 0.46), I² = 0%						

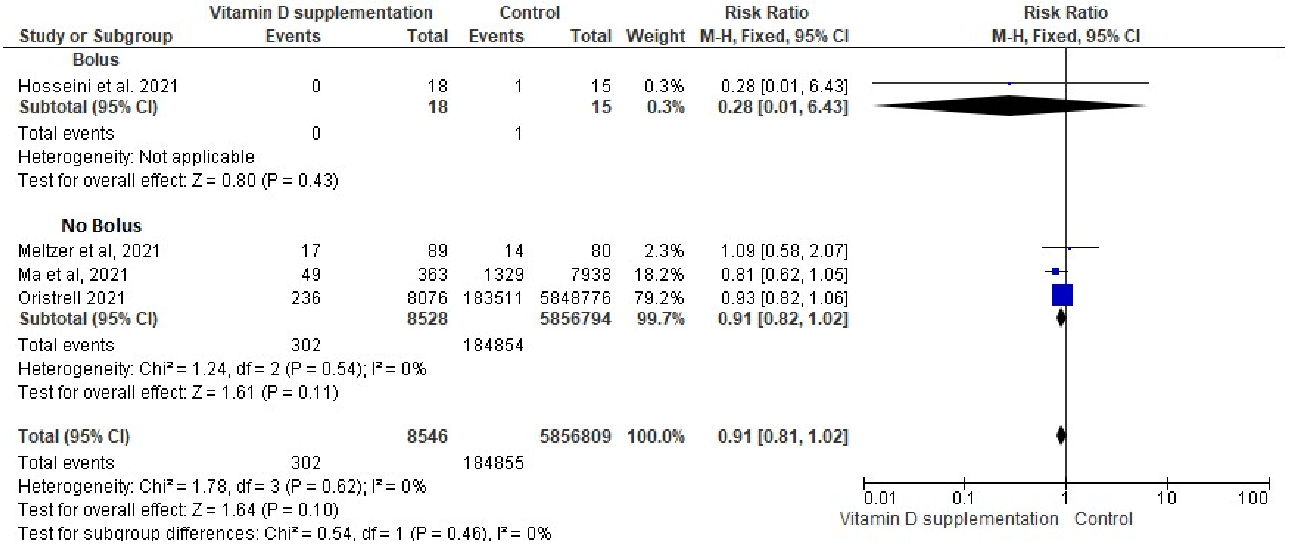

Figure S1. Forest plot of studies investigating the effect of supplementation with vitamin D on the risk of COVID-19 infection by vitamin D regimen (daily or weekly without bolus dosing versus regimen including at least one bolus of 30,000 IU or more). Values are presented as risk ratio with 95% CI determined with the use of Mantel-Haenszel fixed-effects models. Heterogeneity was quantified by I^2 at a significance of $P < 0.10$.

	Vitamin D supplementation		Control			Risk Ratio
**Study or Subgroup**	**Events**	**Total**	**Events**	**Total**	**Weight**	**M-H, Fixed, 95% CI**
**RCTs**						
Hosseini et al. 2021	0	18	1	15	0.3%	0.28 [0.01, 6.43]
**Subtotal (95% CI)**		**18**		**15**	**0.3%**	**0.28 [0.01, 6.43]**
Total events	0		1			
Heterogeneity: Not applicable						
Test for overall effect: Z = 0.80 (P = 0.43)						
**Non-randomized intervention studies**						
Oristrell 2021	236	8076	183511	5848776	99.7%	0.93 [0.82, 1.06]
**Subtotal (95% CI)**		**8076**		**5848776**	**99.7%**	**0.93 [0.82, 1.06]**
Total events	236		183511			
Heterogeneity: Not applicable						
Test for overall effect: Z = 1.11 (P = 0.27)						
**Total (95% CI)**		**8094**		**5848791**	**100.0%**	**0.93 [0.82, 1.05]**
Total events	236		183512			
Heterogeneity: $Chi^2 = 0.56$, df = 1 (P = 0.45); $I^2 = 0\%$						
Test for overall effect: Z = 1.14 (P = 0.25)						
Test for subgroup differences: $Chi^2 = 0.56$, df = 1 (P = 0.45), $I^2 = 0\%$						

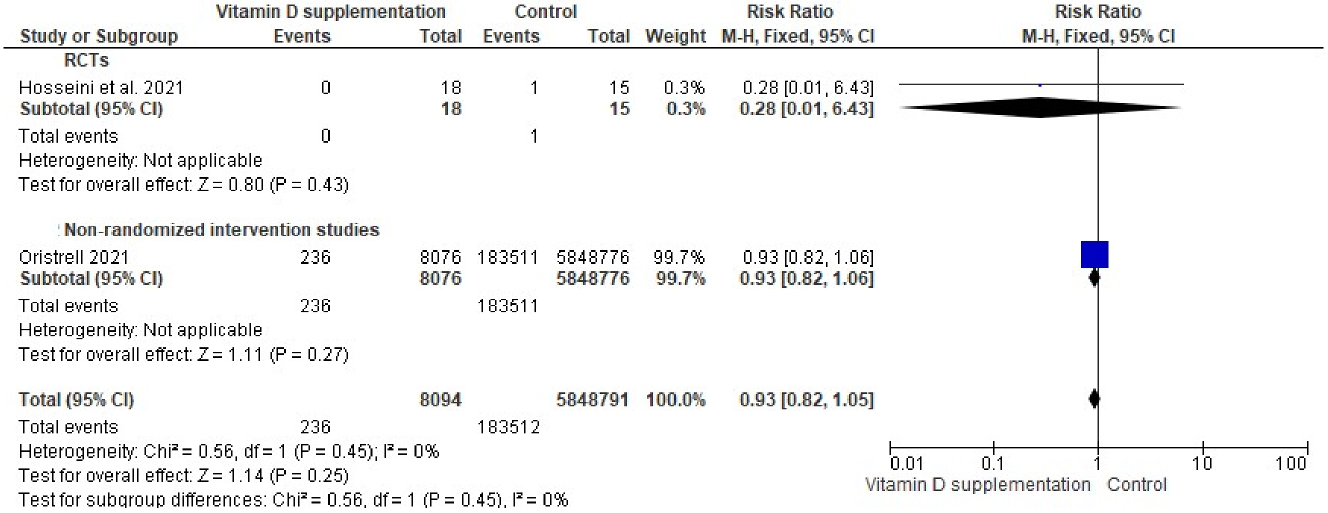

Figure S2. Sensitivity analysis of studies investigating the effects of supplementation with vitamin D on risk of COVID-19 infection. Only studies with a low risk of bias were included. Values are presented as risk ratio with 95% CI determined with the use of Mantel-Haenszel fixed-effects models. Heterogeneity was quantified by I^2 at a significance of P < 0.10.

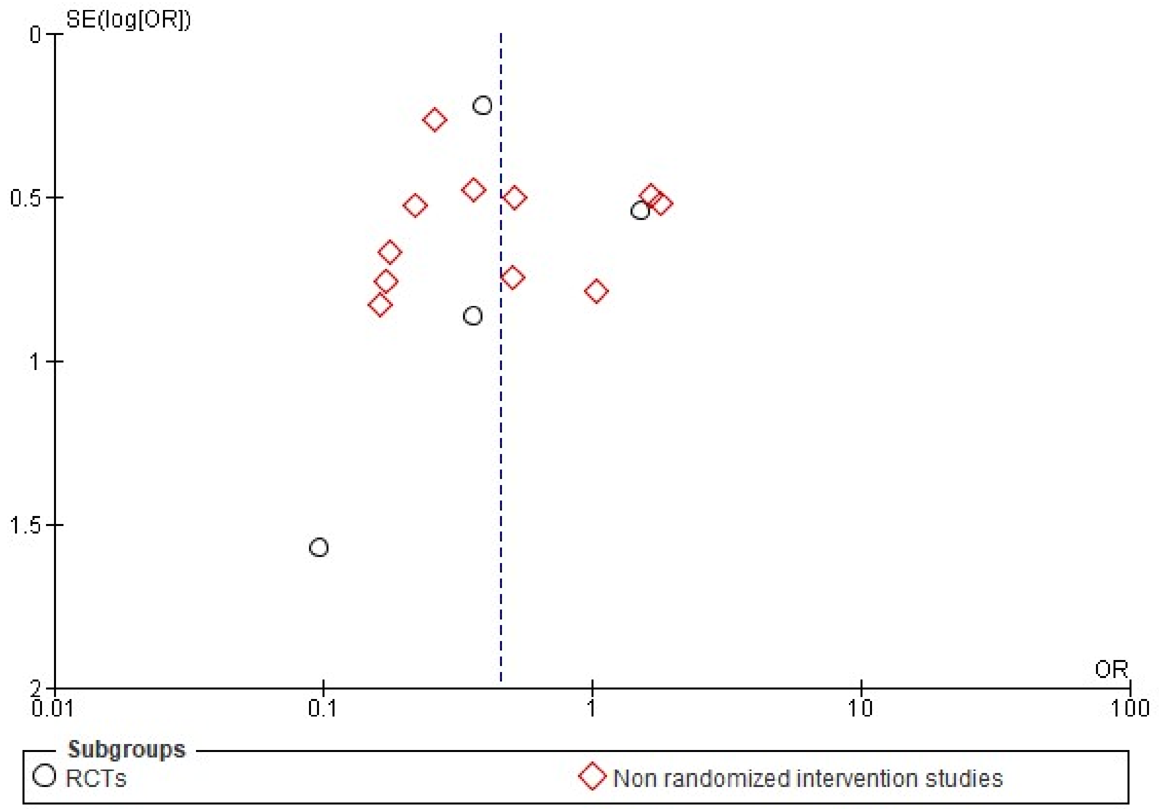

Figure S3. Funnel plot of studies on vitamin D and COVID-19 mortality in randomized clinical trials (circles) and non randomized intervention studies (diamonds)

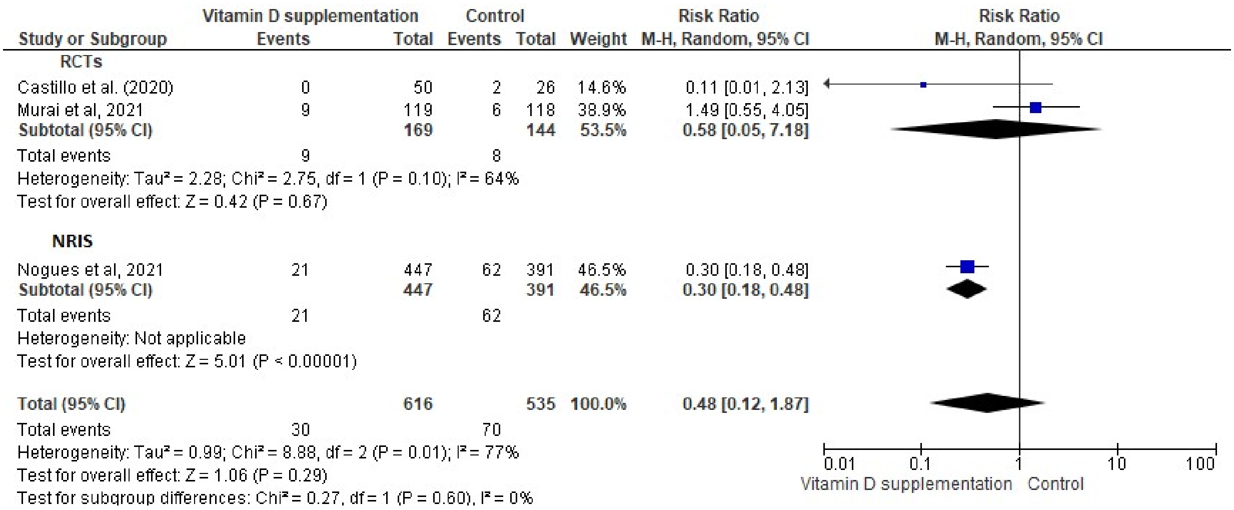

Figure S4. Sensitivity analysis of studies investigating the effects of supplementation with vitamin D on COVID-19 mortality rate. Only studies with a low risk of bias were included. Values are presented as risk ratio with 95% CI determined with the use of Mantel-Haenszel fixed-effects models. Heterogeneity was quantified by I^2 at a significance of $P < 0.10$.

Study or Subgroup	Vitamin D supplementation Events	Total	Control Events	Total	Weight	Risk Ratio M-H, Random, 95% CI
**Bolus**						
Lakkireddy et al. 2021	2	44	5	43	4.2%	0.39 [0.08, 1.91]
Murai et al, 2021	9	119	6	118	7.8%	1.49 [0.55, 4.05]
Guven et al, 2021	9	113	9	62	9.1%	0.55 [0.23, 1.31]
Annweiler et al. 2020 (1)	2	29	10	32	4.8%	0.22 [0.05, 0.92]
Annweiler et al. 2020 (2)	3	16	10	32	6.6%	0.60 [0.19, 1.88]
Giannini et al 2021	11	37	11	54	10.8%	1.46 [0.71, 3.01]
Annweiler et al. 2021 (FU)	16	67	13	28	12.6%	0.51 [0.29, 0.92]
**Subtotal (95% CI)**		**425**		**369**	**56.0%**	**0.69 [0.42, 1.11]**
Total events	52		64			
Heterogeneity: Tau² = 0.17; Chi² = 10.60, df = 6 (P = 0.10); I² = 43%						
Test for overall effect: Z = 1.55 (P = 0.12)						
**No Bolus**						
Castillo et al. (2020)	0	50	2	26	1.4%	0.11 [0.01, 2.13]
Nogues et al 2021, RCT	36	551	57	379	15.3%	0.43 [0.29, 0.65]
Hernandez et al., 2020	2	19	20	197	5.1%	1.04 [0.26, 4.10]
Nogues et al, 2021	21	447	62	391	14.2%	0.30 [0.18, 0.48]
Alcala-Diaz et al.2021	4	79	90	458	8.1%	0.26 [0.10, 0.68]
**Subtotal (95% CI)**		**1146**		**1451**	**44.0%**	**0.37 [0.26, 0.52]**
Total events	63		231			
Heterogeneity: Tau² = 0.03; Chi² = 4.89, df = 4 (P = 0.30); I² = 18%						
Test for overall effect: Z = 5.56 (P < 0.00001)						
**Total (95% CI)**		**1571**		**1820**	**100.0%**	**0.52 [0.36, 0.75]**
Total events	115		295			
Heterogeneity: Tau² = 0.19; Chi² = 23.75, df = 11 (P = 0.01); I² = 54%						
Test for overall effect: Z = 3.48 (P = 0.0005)						
Test for subgroup differences: Chi² = 4.17, df = 1 (P = 0.04), I² = 76.0%						

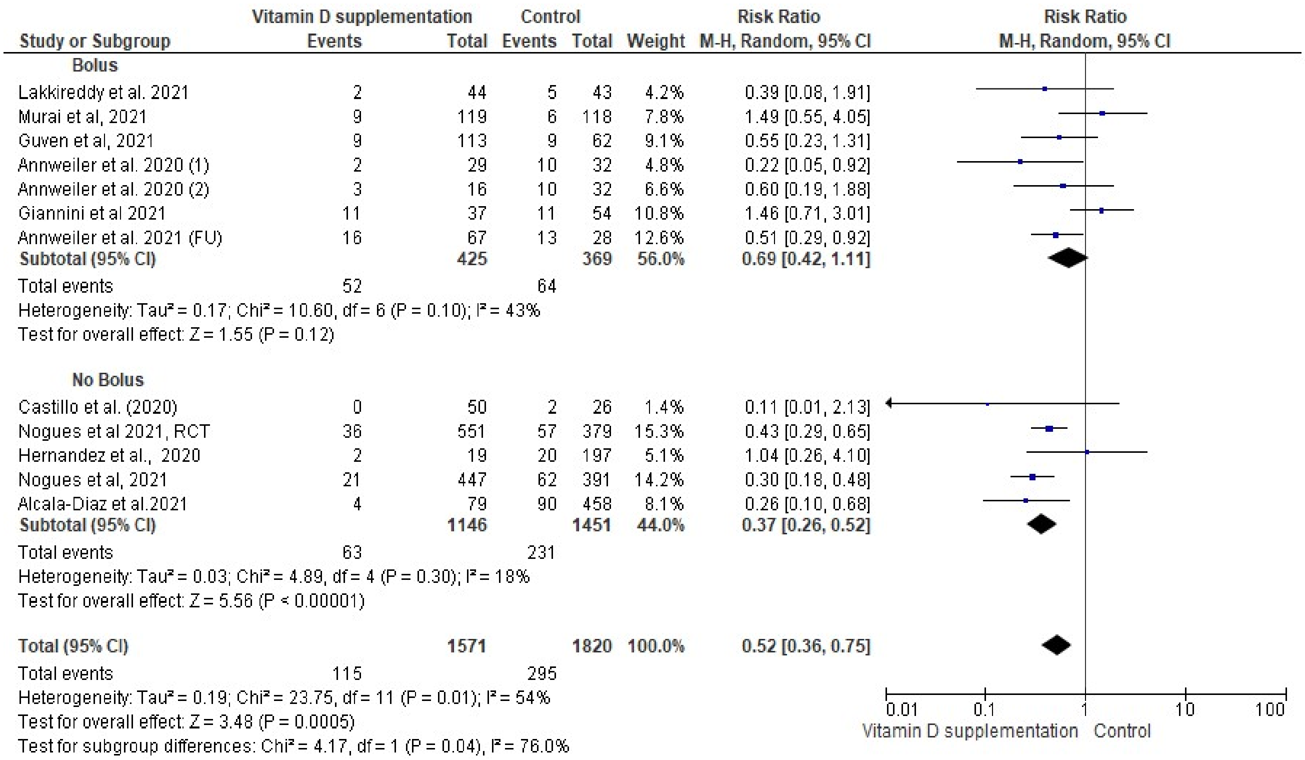

Figure S5. Forest plot of tertiary prevention studies investigating the effects of supplementation with vitamin D on COVID-19 mortality rate by vitamin D regimens (daily or weekly without bolus dosing versus a regimen including at least one bolus of at least 30,000 IU). Values are presented as risk ratio with 95% CI determined with the use of Mantel-Haenszel fixed-effects models. Heterogeneity was quantified by I^2 at a significance of $P < 0.10$.

Study or Subgroup	Vitamin D supplementation Events	Total	Control Events	Total	Weight	Risk Ratio M-H, Random, 95% CI
**25OHD>=25nmol/L**						
Lakkireddy et al. 2021	2	44	5	43	4.2%	0.39 [0.08, 1.91]
Murai et al, 2021	9	119	6	118	7.8%	1.49 [0.55, 4.05]
Nogues et al 2021, RCT	36	551	57	379	15.3%	0.43 [0.29, 0.65]
Annweiler et al. 2021 (FU)	16	67	13	28	12.6%	0.51 [0.29, 0.92]
Hernandez et al., 2020	2	19	20	197	5.1%	1.04 [0.26, 4.10]
Nogues et al, 2021	21	447	62	391	14.2%	0.30 [0.18, 0.48]
**Subtotal (95% CI)**		**1247**		**1156**	**59.2%**	**0.50 [0.33, 0.76]**
Total events	86		163			
Heterogeneity: $Tau^2 = 0.12$; $Chi^2 = 10.14$, df = 5 (P = 0.07); $I^2 = 51\%$						
Test for overall effect: Z = 3.25 (P = 0.001)						
**25OHD<25nmol/L**						
Guven et al, 2021	9	113	9	62	9.1%	0.55 [0.23, 1.31]
Giannini et al 2021	11	37	11	54	10.8%	1.46 [0.71, 3.01]
**Subtotal (95% CI)**		**150**		**116**	**19.9%**	**0.92 [0.35, 2.41]**
Total events	20		20			
Heterogeneity: $Tau^2 = 0.31$; $Chi^2 = 2.89$, df = 1 (P = 0.09); $I^2 = 65\%$						
Test for overall effect: Z = 0.16 (P = 0.87)						
**Not specified**						
Castillo et al. (2020)	0	50	2	26	1.4%	0.11 [0.01, 2.13]
Annweiler et al. 2020 (1)	2	29	10	32	4.8%	0.22 [0.05, 0.92]
Annweiler et al. 2020 (2)	3	16	10	32	6.6%	0.60 [0.19, 1.88]
Alcala-Diaz et al.2021	4	79	90	458	8.1%	0.26 [0.10, 0.68]
**Subtotal (95% CI)**		**174**		**548**	**20.9%**	**0.31 [0.16, 0.60]**
Total events	9		112			
Heterogeneity: $Tau^2 = 0.00$; $Chi^2 = 2.19$, df = 3 (P = 0.53); $I^2 = 0\%$						
Test for overall effect: Z = 3.54 (P = 0.0004)						
**Total (95% CI)**		**1571**		**1820**	**100.0%**	**0.52 [0.36, 0.75]**
Total events	115		295			
Heterogeneity: $Tau^2 = 0.19$; $Chi^2 = 23.75$, df = 11 (P = 0.01); $I^2 = 54\%$						
Test for overall effect: Z = 3.48 (P = 0.0005)						
Test for subgroup differences: $Chi^2 = 3.52$, df = 2 (P = 0.17), $I^2 = 43.2\%$						

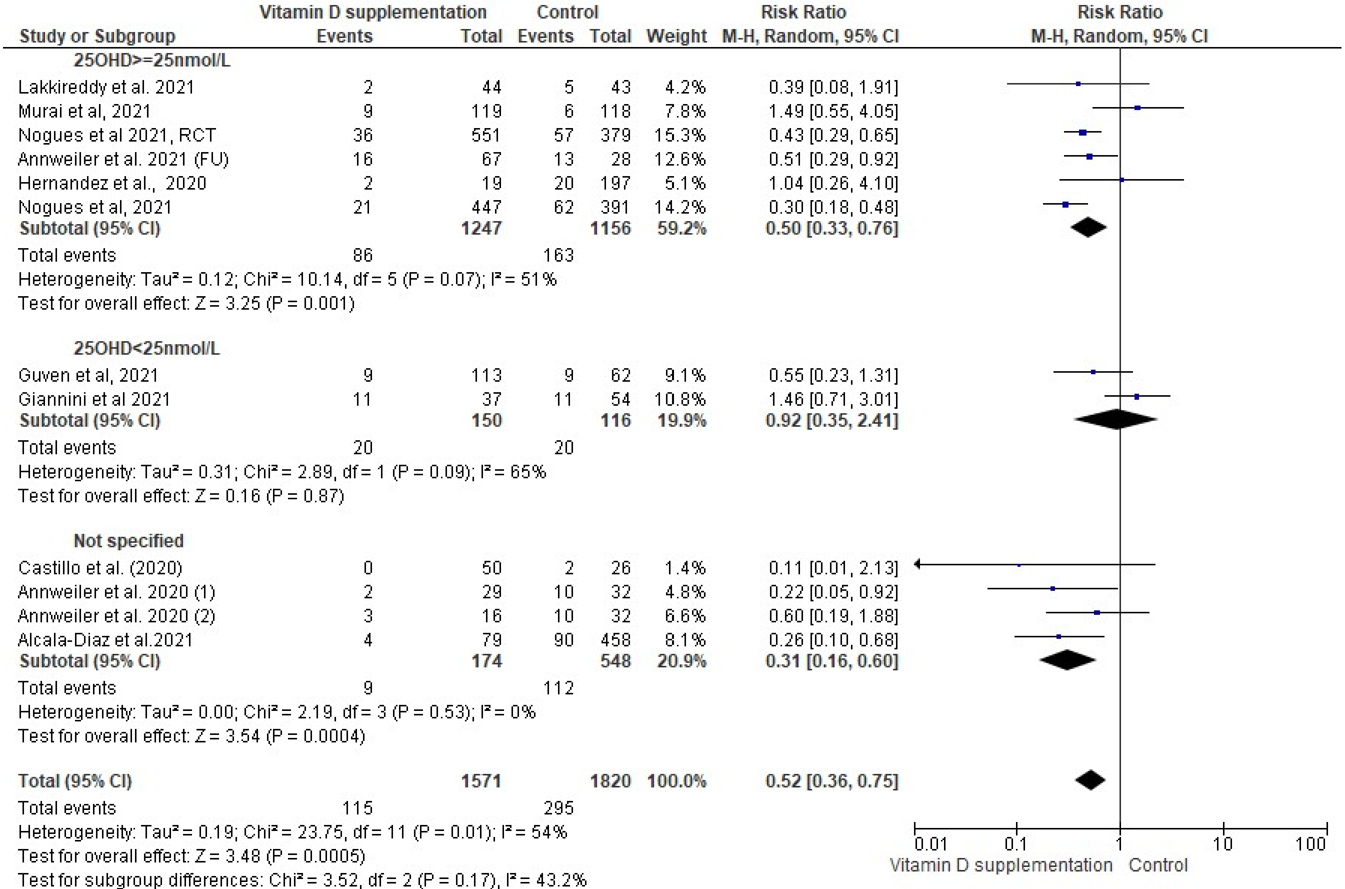

Figure S6. Forest plot of tertiary prevention studies investigating the effects of supplementation with vitamin D on COVID-19 mortality rate by baseline vitamin D status (serum 25-hydroxy level (25(OH)D≥ 25 nmol/L versus 25(OH)D< 25 nmol/L versus not specified). Values are presented as risk ratio with 95% CI determined with the use of Mantel-Haenszel fixed-effects models. Heterogeneity was quantified by I^2 at a significance of P < 0.10.

Study or Subgroup	Vitamin D supplementation Events	Total	Control Events	Total	Weight	Risk Ratio M-H, Random, 95% CI
**Bolus**						
Lakkireddy et al. 2021	2	44	5	43	3.6%	0.39 [0.08, 1.91]
Murai et al, 2021	9	119	6	118	6.3%	1.49 [0.55, 4.05]
Guven et al, 2021	9	113	9	62	7.2%	0.55 [0.23, 1.31]
Annweiler et al. 2020 (1)	2	29	10	32	4.2%	0.22 [0.05, 0.92]
Annweiler et al. 2021	10	57	5	9	7.6%	0.32 [0.14, 0.71]
Annweiler et al. 2020 (2)	3	16	10	32	5.5%	0.60 [0.19, 1.88]
Cangiano et al, 2021	3	20	39	78	5.9%	0.30 [0.10, 0.87]
Giannini et al 2021	11	37	11	54	8.3%	1.46 [0.71, 3.01]
Annweiler et al. 2021 (FU)	16	67	13	28	9.4%	0.51 [0.29, 0.92]
**Subtotal (95% CI)**		**502**		**456**	**57.9%**	**0.57 [0.37, 0.88]**
Total events	65		108			
Heterogeneity: Tau² = 0.20; Chi² = 15.43, df = 8 (P = 0.05); I² = 48%						
Test for overall effect: Z = 2.54 (P = 0.01)						
**No Bolus**						
Castillo et al. (2020)	0	50	2	26	1.3%	0.11 [0.01, 2.13]
Nogues et al 2021, RCT	36	551	57	379	10.8%	0.43 [0.29, 0.65]
Hernandez et al., 2020	2	19	20	197	4.4%	1.04 [0.26, 4.10]
Nogues et al, 2021	21	447	62	391	10.2%	0.30 [0.18, 0.48]
Cereda et al. 2021	7	18	40	152	8.9%	1.48 [0.78, 2.79]
Alcala-Diaz et al.2021	4	79	90	458	6.5%	0.26 [0.10, 0.68]
**Subtotal (95% CI)**		**1164**		**1603**	**42.1%**	**0.50 [0.26, 0.93]**
Total events	70		271			
Heterogeneity: Tau² = 0.39; Chi² = 20.76, df = 5 (P = 0.0009); I² = 76%						
Test for overall effect: Z = 2.17 (P = 0.03)						
**Total (95% CI)**		**1666**		**2059**	**100.0%**	**0.53 [0.37, 0.76]**
Total events	135		379			
Heterogeneity: Tau² = 0.27; Chi² = 37.40, df = 14 (P = 0.0006); I² = 63%						
Test for overall effect: Z = 3.44 (P = 0.0006)						
Test for subgroup differences: Chi² = 0.12, df = 1 (P = 0.73), I² = 0%						

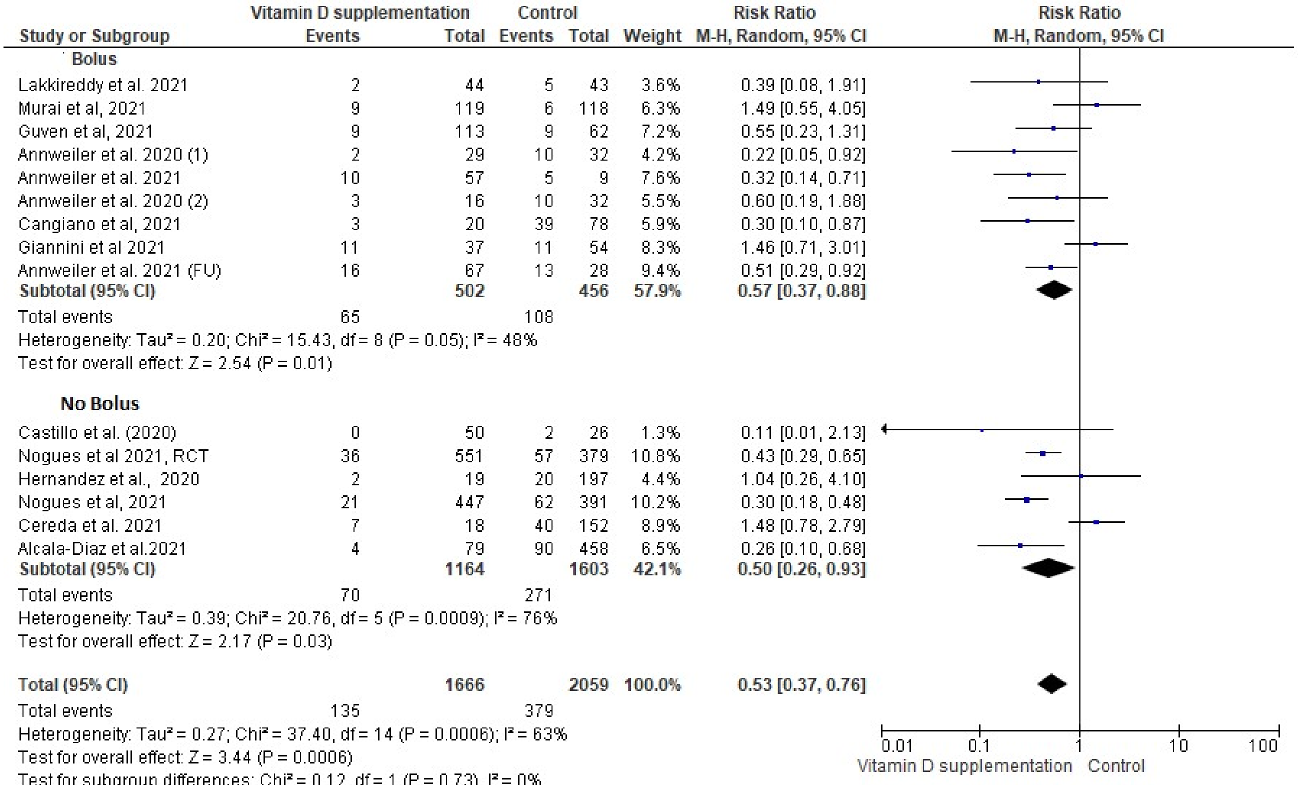

Figure S7. Forest plot of secondary and tertiary prevention studies investigating the effects of supplementation with vitamin D on COVID-19 mortality rate by vitamin D regimens (daily or weekly without bolus dosing versus a regimen including at least one bolus dose of at least 30,000 IU). Values are presented as risk ratio with 95% CI determined with the use of Mantel-Haenszel fixed-effects models. Heterogeneity was quantified by I^2 at a significance of $P < 0.10$.

Study or Subgroup	Vitamin D supplementation Events	Total	Control Events	Total	Weight	Risk Ratio M-H, Random, 95% CI
**25OHD>=25nmol/L**						
Lakkireddy et al. 2021	2	44	5	43	3.6%	0.39 [0.08, 1.91]
Murai et al, 2021	9	119	6	118	6.3%	1.49 [0.55, 4.05]
Nogues et al 2021, RCT	36	551	57	379	10.8%	0.43 [0.29, 0.65]
Annweiler et al. 2021 (FU)	16	67	13	28	9.4%	0.51 [0.29, 0.92]
Hernandez et al., 2020	2	19	20	197	4.4%	1.04 [0.26, 4.10]
Nogues et al, 2021	21	447	62	391	10.2%	0.30 [0.18, 0.48]
Cereda et al. 2021	7	18	40	152	8.9%	1.48 [0.78, 2.79]
**Subtotal (95% CI)**		**1265**		**1308**	**53.6%**	**0.63 [0.38, 1.05]**
Total events	93		203			
Heterogeneity: Tau² = 0.30; Chi² = 22.18, df = 6 (P = 0.001); I² = 73%						
Test for overall effect: Z = 1.76 (P = 0.08)						
**25OHD<25nmol/L**						
Guven et al, 2021	9	113	9	62	7.2%	0.55 [0.23, 1.31]
Giannini et al 2021	11	37	11	54	8.3%	1.46 [0.71, 3.01]
**Subtotal (95% CI)**		**150**		**116**	**15.4%**	**0.92 [0.35, 2.41]**
Total events	20		20			
Heterogeneity: Tau² = 0.31; Chi² = 2.89, df = 1 (P = 0.09); I² = 65%						
Test for overall effect: Z = 0.16 (P = 0.87)						
**Not specified**						
Castillo et al. (2020)	0	50	2	26	1.3%	0.11 [0.01, 2.13]
Annweiler et al. 2020 (1)	2	29	10	32	4.2%	0.22 [0.05, 0.92]
Annweiler et al. 2021	10	57	5	9	7.6%	0.32 [0.14, 0.71]
Annweiler et al. 2020 (2)	3	16	10	32	5.5%	0.60 [0.19, 1.88]
Cangiano et al, 2021	3	20	39	78	5.9%	0.30 [0.10, 0.87]
Alcala-Diaz et al.2021	4	79	90	458	6.5%	0.26 [0.10, 0.68]
**Subtotal (95% CI)**		**251**		**635**	**30.9%**	**0.31 [0.20, 0.49]**
Total events	22		156			
Heterogeneity: Tau² = 0.00; Chi² = 2.19, df = 5 (P = 0.82); I² = 0%						
Test for overall effect: Z = 5.02 (P < 0.00001)						
**Total (95% CI)**		**1666**		**2059**	**100.0%**	**0.53 [0.37, 0.76]**
Total events	135		379			
Heterogeneity: Tau² = 0.27; Chi² = 37.40, df = 14 (P = 0.0006); I² = 63%						
Test for overall effect: Z = 3.44 (P = 0.0006)						
Test for subgroup differences: Chi² = 6.32, df = 2 (P = 0.04), I² = 68.4%						

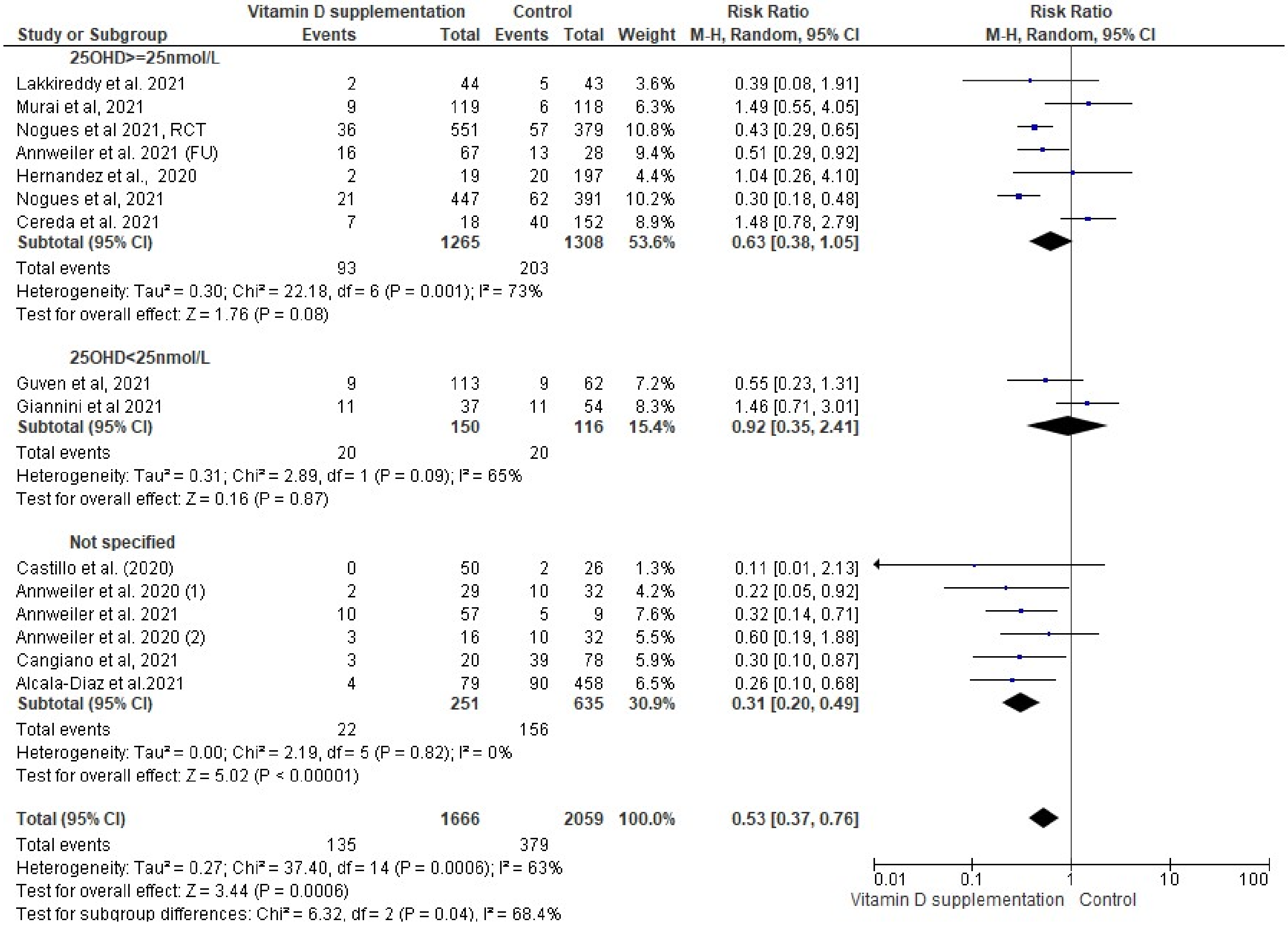

Figure S8. Forest plot of secondary and tertiary prevention studies investigating the effects of supplementation with vitamin D on COVID-19 mortality rate by baseline vitamin D status (serum 25-hydroxy level (25(OH)D≥ 25 nmol/L versus 25(OH)D< 25 nmol/L versus not specified). Values are presented as risk ratio with 95% CI determined with the use of Mantel-Haenszel fixed-effects models. Heterogeneity was quantified by I^2 at a significance of $P < 0.10$.

Study or Subgroup	Vitamin D supplementation Events	Total	Control Events	Total	Weight	Risk Ratio M-H, Random, 95% CI
**RCTs**						
Castillo et al. (2020)	1	50	13	26	20.4%	0.04 [0.01, 0.29]
Murai et al, 2021	19	119	25	118	39.4%	0.75 [0.44, 1.29]
**Subtotal (95% CI)**		**169**		**144**	**59.8%**	**0.20 [0.01, 4.26]**
Total events	20		38			
Heterogeneity: Tau² = 4.37; Chi² = 9.00, df = 1 (P = 0.003); I² = 89%						
Test for overall effect: Z = 1.03 (P = 0.30)						
**NRIS**						
Nogues et al, 2021	20	447	82	391	40.2%	0.21 [0.13, 0.34]
**Subtotal (95% CI)**		**447**		**391**	**40.2%**	**0.21 [0.13, 0.34]**
Total events	20		82			
Heterogeneity: Not applicable						
Test for overall effect: Z = 6.45 (P < 0.00001)						
**Total (95% CI)**		**616**		**535**	**100.0%**	**0.25 [0.07, 0.86]**
Total events	40		120			
Heterogeneity: Tau² = 0.93; Chi² = 17.36, df = 2 (P = 0.0002); I² = 88%						
Test for overall effect: Z = 2.20 (P = 0.03)						
Test for subgroup differences: Chi² = 0.00, df = 1 (P = 0.97), I² = 0%						

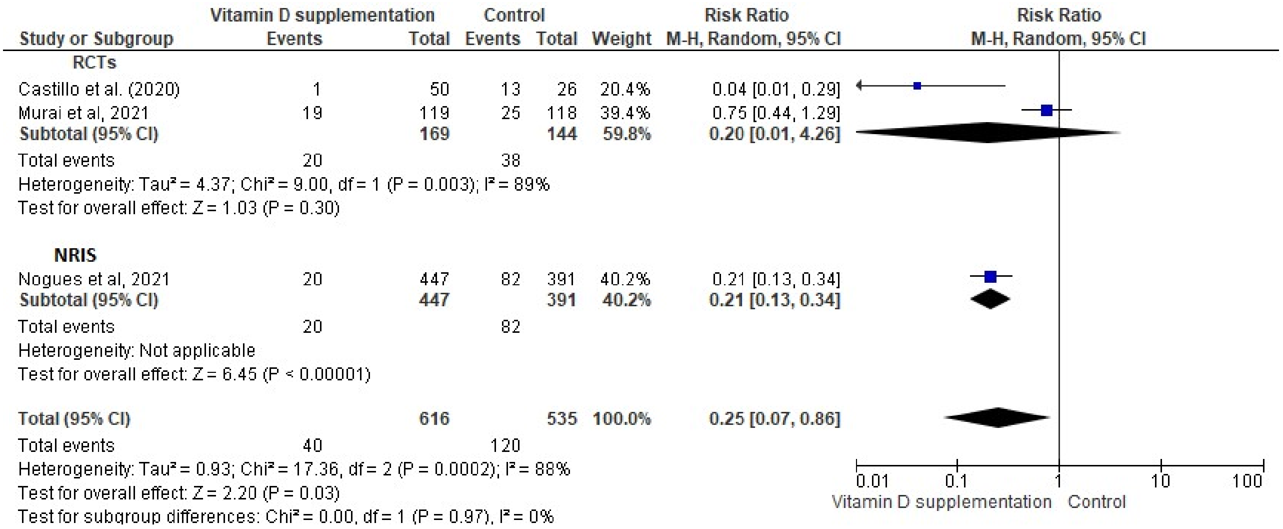

Figure S9. Sensitivity analysis of studies investigating the effects of supplementation with vitamin D on COVID-19 associated ICU admission rate. Only studies with a low risk of bias were included. Values are presented as risk ratio with 95% CI determined with the use of Mantel-Haenszel fixed-effects models. Heterogeneity was quantified by I^2 at a significance of $P < 0.10$.

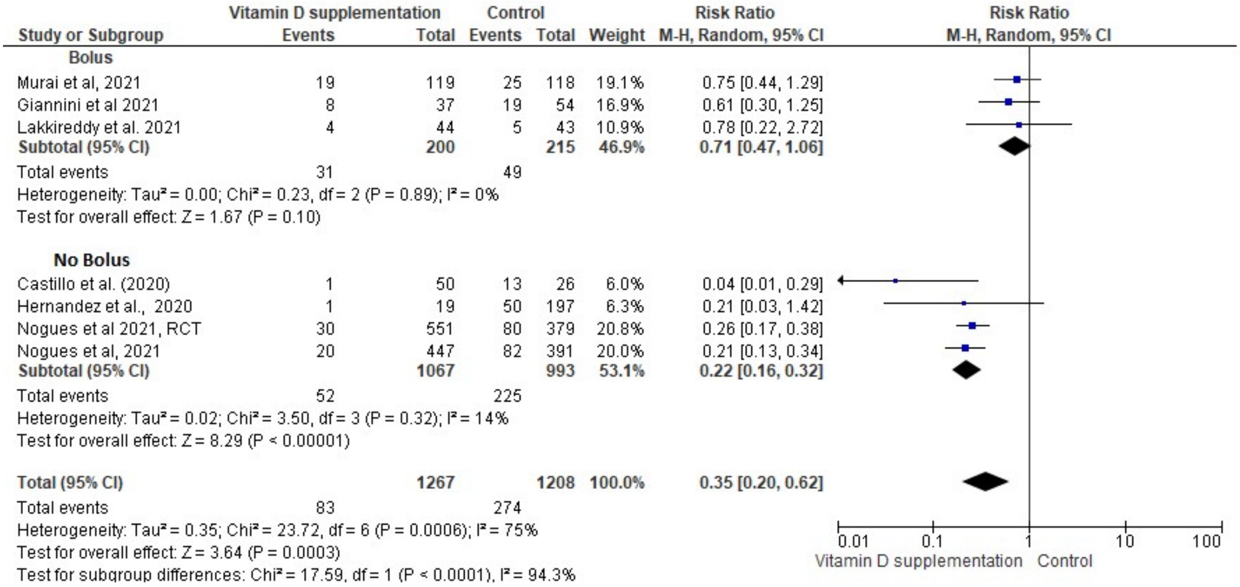

Figure S10. Forest plot of studies investigating the effects of supplementation with vitamin D on COVID-19 associated ICU admission rate by vitamin D regimens (daily or weekly without bolus dosing versus a regimen including at least one bolus dose of at least 30,000 IU). Values are presented as risk ratio with 95% CI determined with the use of Mantel-Haenszel fixed-effects models. Heterogeneity was quantified by I^2 at a significance of P < 0.10.

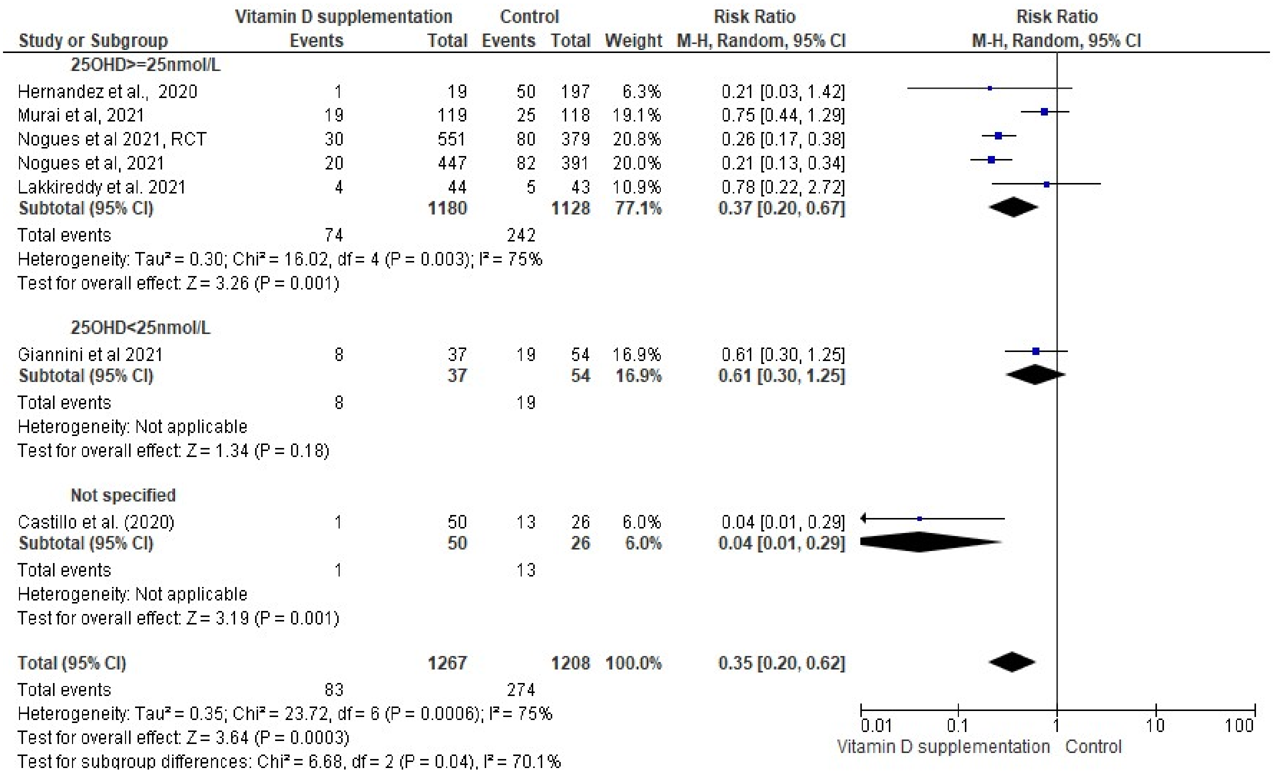

Figure S11. Forest plot of studies investigating the effects of supplementation with vitamin D on COVID-19 associated ICU admission rate by baseline vitamin D status (serum 25-hydroxy level (25(OH)D≥ 25 nmol/L versus 25(OH)D< 25 nmol/L versus not specified) in tertiary prevention studies. Values are presented as risk ratio with 95% CI determined with the use of Mantel-Haenszel fixed-effects models. Heterogeneity was quantified by I^2 at a significance of $P < 0.10$.

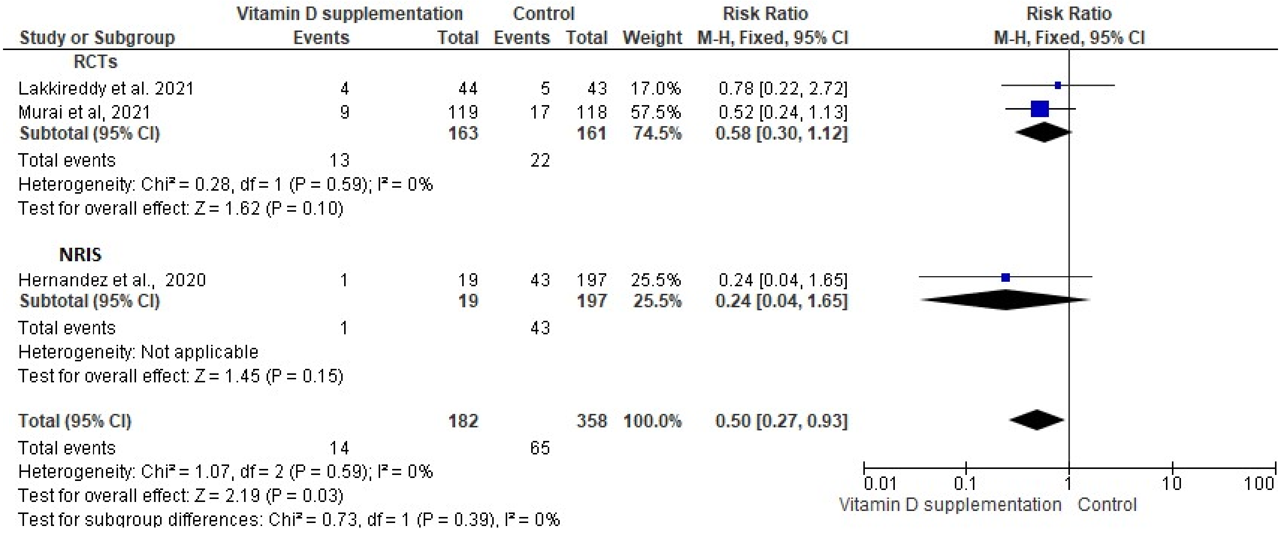

Figure S12. Forest plot of studies investigating the effects of supplementation with vitamin D on the need for ventilation in patients with COVID-19 by study design. Values are presented as risk ratio with 95% CI determined with the use of Mantel-Haenszel fixed-effects models. Heterogeneity was quantified by I^2 at a significance of $P < 0.10$.

Study or Subgroup	Vitamin D supplementation Mean	SD	Total	Control Mean	SD	Total	Weight	Mean Difference IV, Fixed, 95% CI
**RCTs**								
Lakkireddy et al. 2021	13	5	44	14	5	43	1.2%	-1.00 [-3.10, 1.10]
Murai et al, 2021	7	1	120	7	1.3	120	60.3%	0.00 [-0.29, 0.29]
**Subtotal (95% CI)**			**164**			**163**	**61.4%**	**-0.02 [-0.31, 0.27]**
Heterogeneity: Chi² = 0.85, df = 1 (P = 0.36); I² = 0%								
Test for overall effect: Z = 0.13 (P = 0.90)								
**NRIS**								
Abdulateef et al 2021	9	1.66	127	9	2	300	38.6%	0.00 [-0.37, 0.37]
**Subtotal (95% CI)**			**127**			**300**	**38.6%**	**0.00 [-0.37, 0.37]**
Heterogeneity: Not applicable								
Test for overall effect: Z = 0.00 (P = 1.00)								
**Total (95% CI)**			**291**			**463**	**100.0%**	**-0.01 [-0.24, 0.22]**
Heterogeneity: Chi² = 0.86, df = 2 (P = 0.65); I² = 0%								
Test for overall effect: Z = 0.10 (P = 0.92)								
Test for subgroup differences: Chi² = 0.01, df = 1 (P = 0.94), I² = 0%								

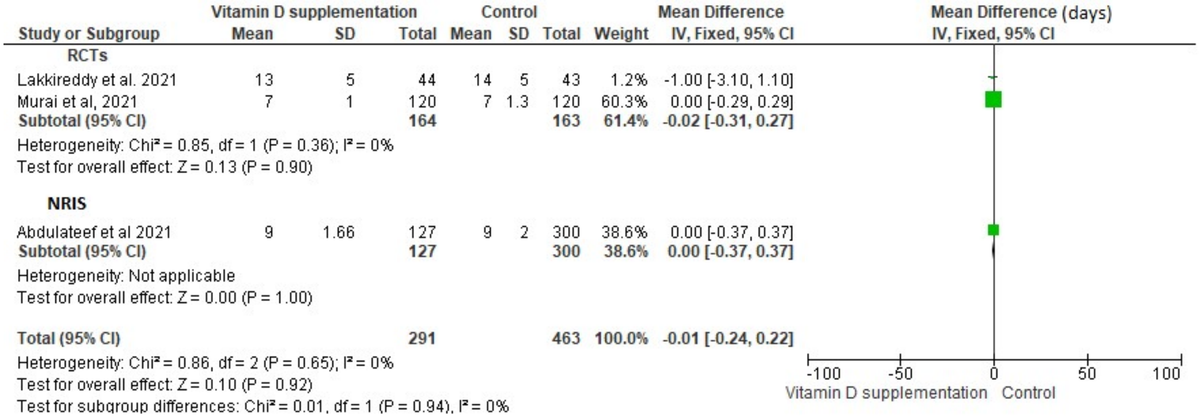

Figure S13. Forest plot of studies investigating the effects of supplementation with vitamin D on the length of hospitalization (days) in patients with COVID-19 by study design. Values are presented as mean difference with 95% CI determined with the use of Mantel-Haenszel fixed-effects models. Heterogeneity was quantified by I^2 at a significance of $P < 0.10$.
